# Seasonal variation in D2/3 dopamine receptor availability in the human brain

**DOI:** 10.1007/s00259-024-06715-9

**Published:** 2024-05-11

**Authors:** Lihua Sun, Tuulia Malén, Jouni Tuisku, Valtteri Kaasinen, Jarmo A. Hietala, Juha Rinne, Pirjo Nuutila, Lauri Nummenmaa

**Affiliations:** 1 Huashan Institute of Medicine, Huashan Hospital, Fudan University, Shanghai, China; 2 Turku PET Centre, University of Turku, Turku, Finland; 3 Turku PET Centre, Turku University Hospital, Turku, Finland; 4 Clinical Neurosciences, University of Turku, Turku, Finland; 5 Turku University Hospital, Neurocenter, Turku, Finland; 6 Department of Psychiatry, University of Turku, Turku University Hospital, Turku, Finland; 7 Department of Endocrinology, Turku University Hospital, Turku, Finland; 8 Department of Psychology, University of Turku, Turku, Finland

**Keywords:** Dopamine D2 receptor, Seasonality, Striatum, Caudate, Positron emission tomography, Reward

## Abstract

**Purpose:**

Brain functional and physiological plasticity is essential to combat dynamic environmental challenges. The rhythmic dopamine signaling pathway, which regulates emotion, reward and learning, shows seasonal patterns with higher capacity of dopamine synthesis and lower number of dopamine transporters during dark seasons. However, seasonal variation of the dopamine receptor signaling remains to be characterized.

**Methods:**

Based on a historical database of healthy human brain [^11^C]raclopride PET scans (*n* = 291, 224 males and 67 females), we investigated the seasonal patterns of D2/3 dopamine receptor signaling. Daylength at the time of scanning was used as a predictor for brain regional non-displaceable binding of the radiotracer, while controlling for age and sex.

**Results:**

Daylength was negatively correlated with availability of D2/3 dopamine receptors in the striatum. The largest effect was found in the left caudate, and based on the primary sample, every 4.26 h (i.e., one standard deviation) increase of daylength was associated with a mean 2.8% drop (95% CI -0.042 to -0.014) of the receptor availability.

**Conclusions:**

Seasonally varying D2/3 receptor signaling may also underlie the seasonality of mood, feeding, and motivational processes. Our finding suggests that in future studies of brain dopamine signaling, especially in high-latitude regions, the effect of seasonality should be considered.

**Supplementary Information:**

The online version contains supplementary material available at 10.1007/s00259-024-06715-9.

## Introduction

Brain functional and physiological plasticity is essential to combat dynamic environmental challenges, for survival and the wellbeing. This includes the potential seasonal patterns of dopamine signaling. For instance, in vivo imaging data in both healthy subjects [1] and patients [2] show increase of striatal dopamine synthesis in fall and winter. Dopamine transporter binding in the left caudate is lowered during dark seasons [3]. Also, preclinical studies suggest that longer photoperiod stimulates nucleus accumbens dopamine release in female mice [4]. Dopamine signaling plays an important role in seasonal breeding of animals [5] and, via interaction with the melatonin signaling, it affects circadian rhythms [6, 7]. Further, dopamine regulates emotion, reward, and learning that demonstrate seasonal patterns, with learning ability, attention and positive emotions all at low levels during winter months [8–10]. Human feeding behavior, where the brain dopamine signaling also plays a crucial role [11], similarly demonstrates seasonal patterns with increased caloric intake of fats in fall or winter [12]. Detailed knowledge on how dopamine signaling adapts to seasonal rhythms is not only essential for understanding normal molecular brain plasticity, but also crucial in understanding psychiatric conditions with seasonally varying onset and severity, such as the seasonal affective disorders.

Brain dopamine signaling is primarily relayed through the D1 family and D2 family receptors (D2Rs), where the D2Rs are found with both post- and pre-synaptic expression. This indicates an additional role of D2Rs in receptor-mediated feedback control in dopamine signaling. D2Rs are involved in both presynaptic dopamine release [13] and in regulation of dopamine synthesis [14]. For instance, D2R agonist inhibits tyrosine hydroxylase (TH) function, where TH action on L-DOPA is a rate-limiting step for dopamine synthesis [15]. Also, cAMP is known to induce expression of TH, and D2Rs inhibit the cAMP signaling [16]. Further, D2Rs may exhibit complementary interaction with dopamine transporter, so that D2Rs enhance the velocity of dopamine synaptic reuptake [17, 18]. Therefore, rhythmic D2R functions potentially direct all steps of dopamine signaling including its presynaptic synthesis and release, and extracellular clearance. Yet, potential seasonal patterns of D2R signaling remains to be characterized.

**Fig. 1:**
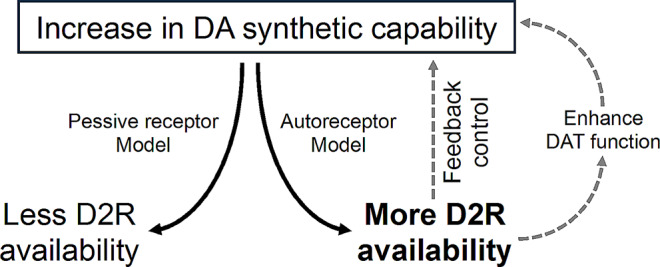
The autoreceptor model of D2/D3 receptor (D2R) availability considering increased dopamine (DA) synthetic capability and reduced dopamine transporter availability (DAT) during dark seasons

Here we analyzed a large historical dataset of healthy subjects’ brain PET scans (*n* = 291) measuring D2Rs (i.e., primarily the D2/3 receptors) with antagonist radioligand [^11^C]raclopride. We focused on the striatal and thalamic regions where this receptor is highly expressed and can be reliably measured with the used radioligand [19, 20]. Daylength was used as a regressor to predict the regional D2R availability, and data were analyzed separately for left and right hemispheres. Prior findings show that dark seasons are associated with enhanced dopamine synthetic ability [1, 2] and also reduced amount of dopamine transporters [3]. This may indicate reduced amount of extracellular dopamine, despite of increased synthesis, and enhanced presynaptic control of dopamine release. Therefore, considering the autoreceptor role of D2Rs in a feedback control, we hypothesized short daylength to be associated with increased striatal D2R availability, Fig. 1. A preprint version of the manuscript was originally reposited to bioRxiv.

## Methods

### Data

**Fig. 2:**
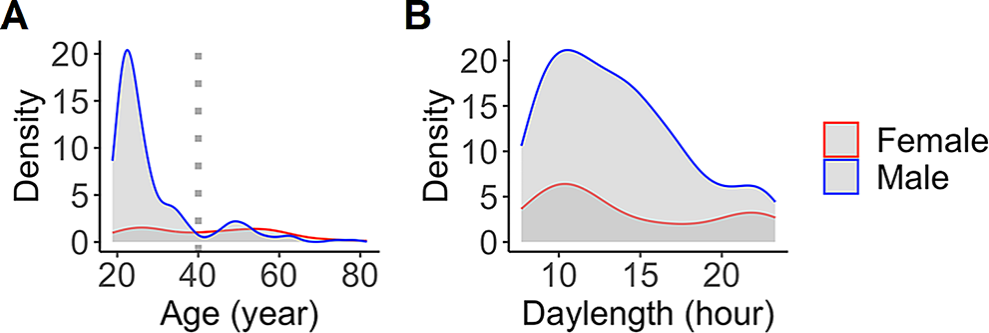
Sex-specific distributions of age (**A**) and local daylength at the time of scanning (**B**). Dotted line shows the age cutoff (40 years) for the primary sample

The data were 291 baseline [^11^C]raclopride scans from healthy control subjects (224 males and 67 females; age 19–81 years) collected at Turku PET Centre between 2004 and 2018. Distribution of local daylength at the time of the scan and age are illustrated in Fig. 2. For each subject, daylength was calculated as the daytime plus civil twilight on the day when the PET image was acquired, based on geographic location of the Turku PET Center (Turku, Finland; latitude = 60.4518; longitude = 22.2666), as previously [21].

### PET data acquisition and image processing

Antagonist radioligand [^11^C]raclopride binds to D2Rs, allowing reliable quantification of striatal and thalamic D2R availability [22]. Detailed information regarding technical properties of scanners and tracer injection information is found in supplementary Table S1 & S2. We included the following regions of interest (ROIs) as delineated based on the AAL atlas [23]: nucleus accumbens, caudate nucleus (caudate), putamen and thalamus, which were separately analyzed for left and right hemispheres.

Preprocessing was done using the Magia toolbox [24]. Tracer binding was quantified using the outcome measure binding potential (*BP*_*ND*_), which is the ratio of specific binding to non-displaceable binding in tissue [25]. *BP*_*ND*_ was estimated using a simplified reference tissue model (SRTM) [26] with cerebellar gray matter as the reference region. Acquisition length was harmonized by including first 52 min from each scan [20], independently of scan duration.

### Statistical analysis

Data were analyzed using linear mixed effects regression with the R statistical software (version 4.3.0) and the lme4 package. Because ageing influences D2R availability [27–29], primary analysis was based on subjects younger than 40 years old (primary analysis, *n* = 227). Findings based on the whole sample (*n* = 291) are presented in the supplementary data.

D2R *BP*_*ND*_ was modelled separately for each region of interest using fixed factors including daylength at scanning, age and sex; scanner types were used as the random intercept. D2R *BP*_*ND*_ were log-transformed, while daylength and age were standardized in the statistical models. Because BMI is only weakly associated with *BP*_*ND*_ [29] and a large portion of the subjects lacked BMI data, it was excluded from the statistical models.

### Voxel-level analysis

The data were analyzed at the voxel level using SPM12 (Wellcome Trust Center for Imaging, London, UK, http://www.fil.ion.ucl.ac.uk/spm). The normalized *BP*_*ND*_ images were entered into general linear models, where they were predicted with daylength. Age, sex and scanner types were entered into the models as nuisance covariates. Because [^11^C]raclopride binds selectively only in striatum and thalamus, the analysis was restricted to the high-binding sites by creating a single mask covering these regions (caudate, putamen, nucleus accumbens and thalamus). Statistical threshold was set at *p* < 0.05, FDR-corrected at cluster level.

## Results

### Regional analysis in the primary sample

The primary sample (*n* = 227) included 195 males and 32 females. The mean age was 25.6 y (SD = 4.87 y, range = 18.82–39.47 y) and average daylength exposure was 13.61 h (SD = 4.26 h, range = 7.7–23.25 h). More detailed information of the primary sample is found in supplementary Table S3.

**Fig. 3:**
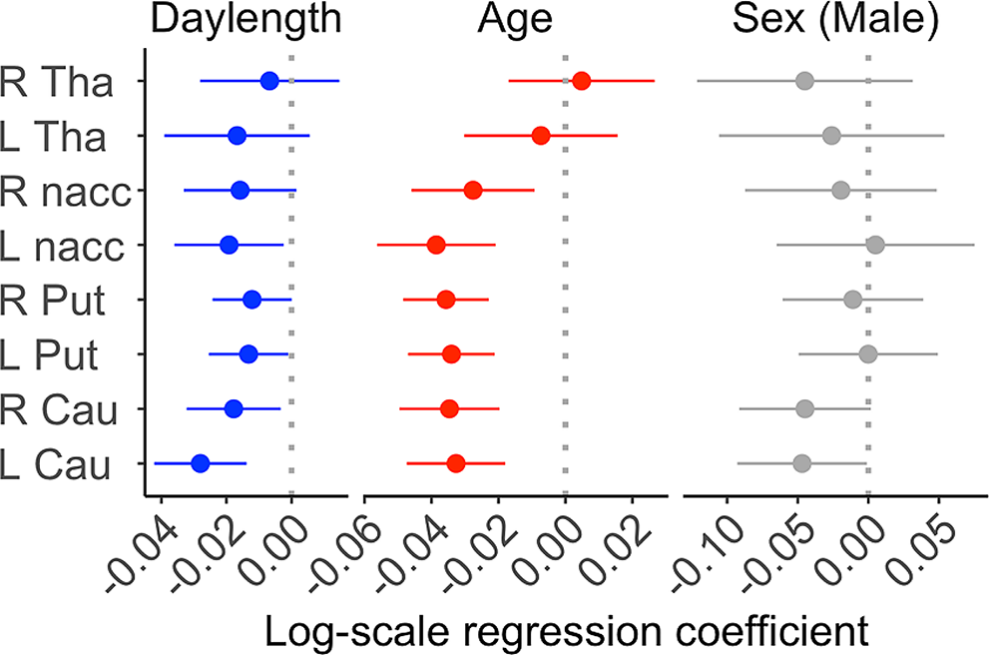
Effect sizes (i.e., point estimate and the 95% confidence interval) of daylength, age and sex (male) on D2R BP_ND_ in different brain regions. L = Left, R = Right, Cau = Caudate, Put = Putamen, nacc = Nucleus accumbens, Tha = Thalamus

**Fig. 4:**
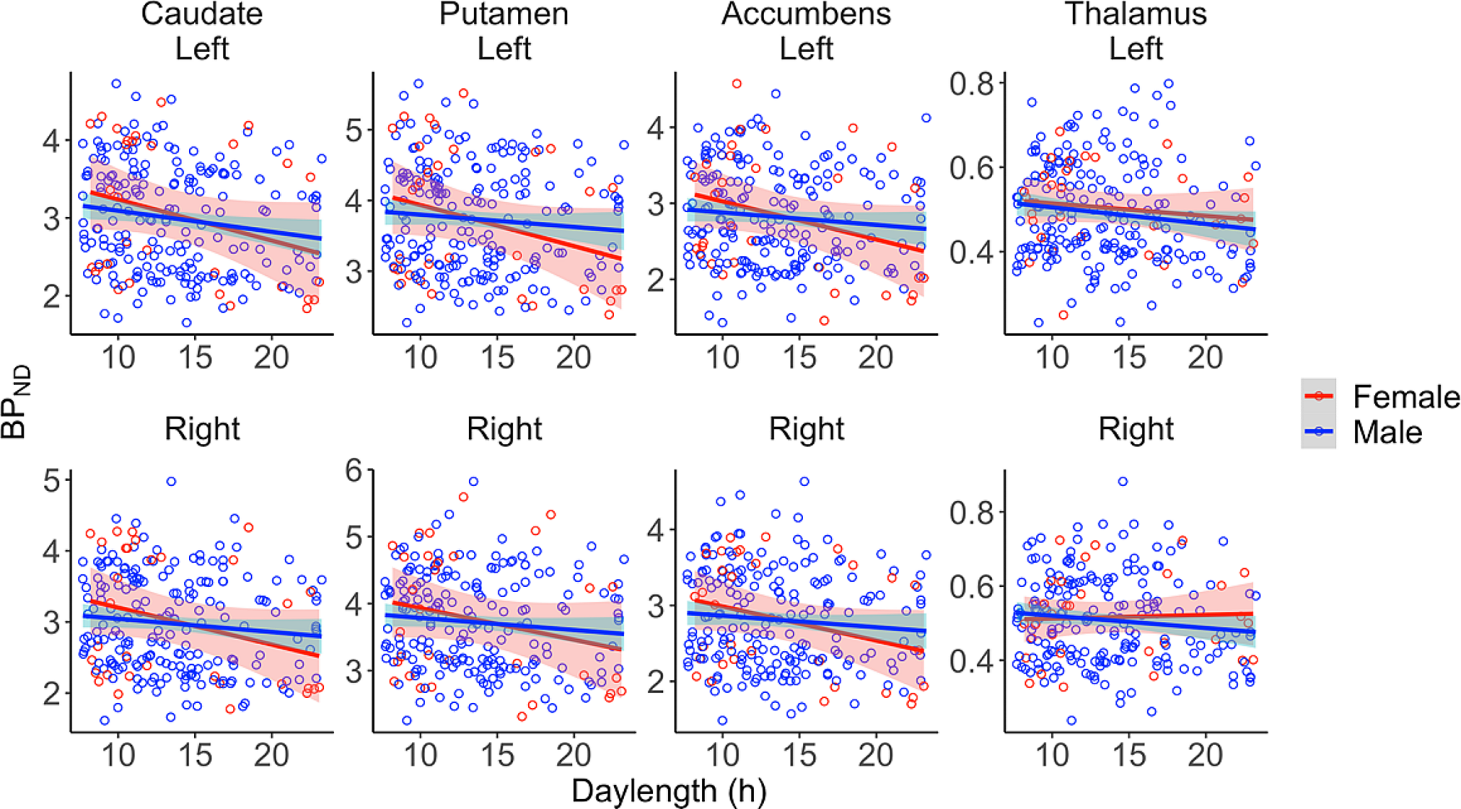
Association between regional D2R BP_ND_ and daylength in each ROI, separately for males and females. Red and blue lines show Least Squares regression lines, and their 95% confidence intervals are shaded

Data revealed that daylength was linearly and negatively associated with regional D2R availability (*BP*_*ND*_) in most regions (Table 1; Fig. 3**&** Fig. 4**)**. Age was negatively associated with D2R *BP*_*ND*_, and male subjects had lower D2R *BP*_*ND*_ (Fig. 3) than females. Information of the full sample and the corresponding findings are found in supplementary Table S4 & S5.

**Table 1:** Effect of daylength on regional D2R BP_ND_ in the brain (uncorrected for multiple comparison)

Hemisphere	Region	Beta	95% CI	t	p
Left	Caudate	-0.028	-0.042, -0.014	-3.86	0.00015***
Right	Caudate	-0.018	-0.032, -0.0033	-2.41	0.017*
Left	Putamen	-0.013	-0.025, -0.001	-2.11	0.036*
Right	Putamen	-0.012	-0.024, -0.000006	-1.95	0.053*
Left	NACC	-0.019	-0.036, -0.0024	-2.23	0.027*
Right	NACC	-0.016	-0.033, 0.0015	-1.78	0.077
Left	Thalamus	-0.016	-0.039, 0.0056	-1.46	0.15
Right	Thalamus	-0.0065	-0.028, 0.015	-0.60	0.55

We compared the effect size (i.e., 95% CI) between daylength, age and sex, Fig. 3. In the primary sample, every 4.26 h (i.e., one standard deviation) increase of daylength was associated with a mean 2.8% drop of D2R *BP*_*ND*_ in the left caudate. In the same region, every 4.87 y (i.e., one standard deviation) increase of age was associated with a mean 3.3% drop of D2R *BP*_*ND*_. Corresponding results based on the full sample are found in Supplementary Figure S1.

### Voxel-level analysis in the primary sample

**Fig. 5:**
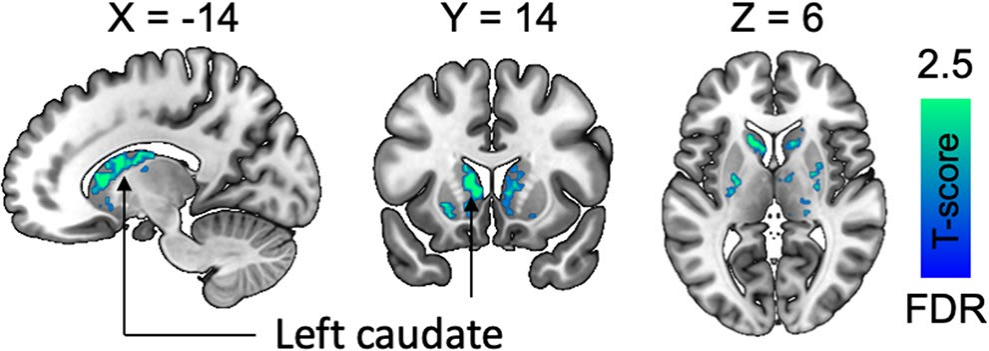
Daylength was as significant predictor for D2R BP_ND_ in the striatum. Data are thresholded at *P* < 0.05 with false discovery rate (FDR) cluster-level correction

We next run a complementary voxel-level analysis for the high-binding regions (striatum and thalamus). Similar as in the ROI analysis, daylength was found to be a significant predictor for striatal D2R *BP*_*ND*_, Fig. 5.

### Effect of daylength in the whole sample

Considering the impact of ageing on dopamine signaling [27–29], there is always a tradeoff between sample size and the allowed maximum age. In addition to a primary sample analysis, we also investigated how the effect of daylength survived a stepwise minimization of sample in accord to the allowed maximum age.

**Fig. 6:**
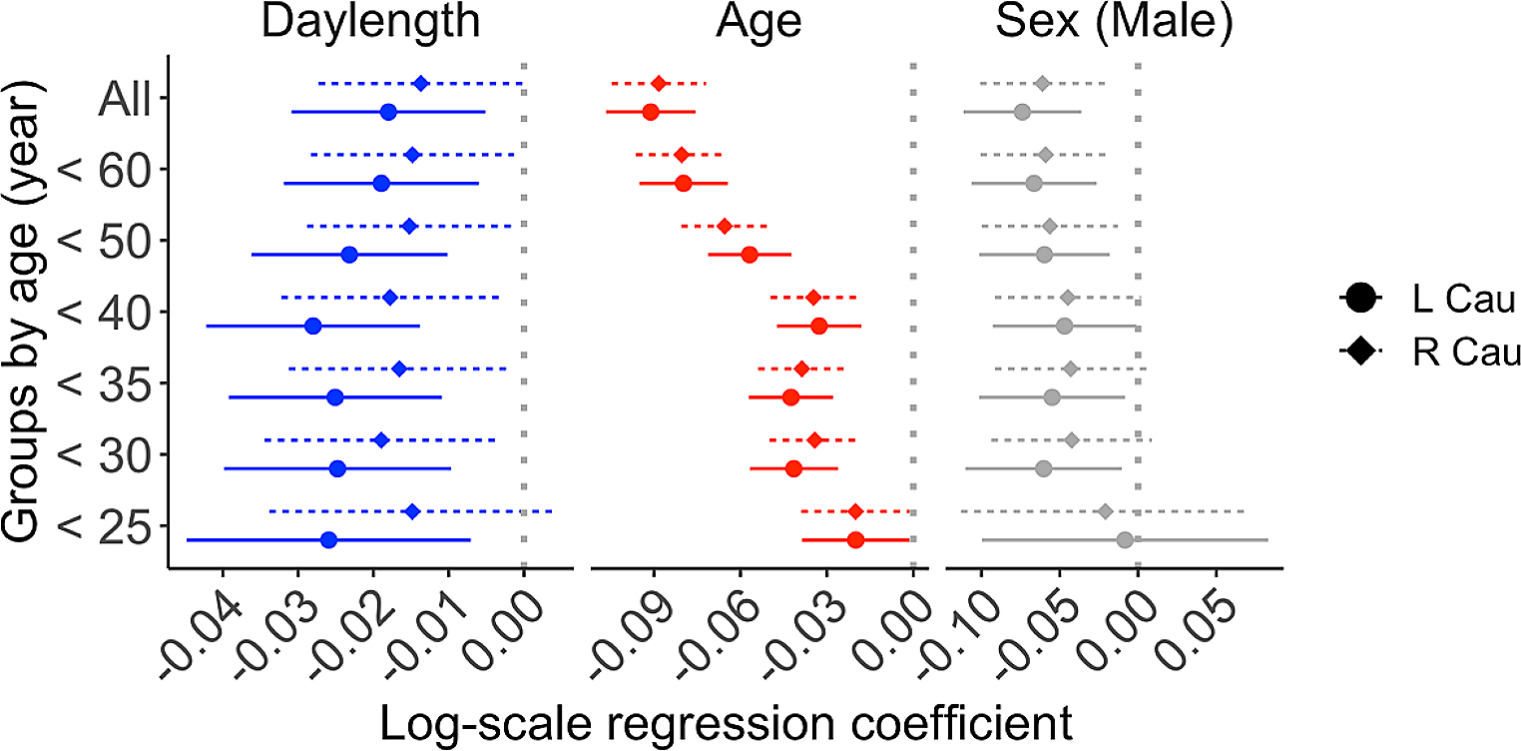
Effect sizes for daylength, age and sex (male) on D2R BP_ND_ in the left and right caudate in different groups defined by maximum age. Plots of other ROIs are found in the supplementary Figure S2

We analysed the effect of daylength in different groups where the whole sample was divided into 7 groups in accord to the allowed maximum age, Table 2. Results showed that the effect of daylength of D2R *BP*_*ND*_ retained across different groups, especially in the left caudate (Fig. 6 and Supplementary Figure S2). This accords with previous findings that highlight the importance of the left caudate region, regarding both seasonal variation of dopamine transporter signaling [3] and dopamine-relevant etiology in seasonal affective disorders [30].

**Table 2:** Daylength and age across groups defined by maximum age. Information of the primary sample are highlighted in bold

Groups	Number of subjects (female)	Age (y)			Daylength			
		mean	SD		mean	SD	min	max
All	291(67)	31.65	13.43		13.84	4.43	7.70	23.28
< 60 y	279(61)	30.07	11.19		13.73	4.40	7.70	23.28
< 50 y	252(43)	27.38	7.92		13.66	4.35	7.70	23.25
**< 40 y**	**227(32)**	**25.26**	**4.87**		**13.61**	**4.26**	**7.70**	**23.25**
< 35 y	213(27)	24.45	3.79		13.53	4.20	7.70	23.25
< 30 y	188(23)	23.41	2.6		13.44	4.17	7.70	23.25
< 25 y	135(10)	22.07	1.57		13.14	4.10	7.70	23.25

## Discussion

Our main finding was that daylength modulated in vivo brain D2R availability in healthy humans. Specifically, striatal D2R availability was elevated in autumn-winter time when the days are short and lowered during the spring-summer time when the days are long. This finding is based on, to our knowledge, the largest database of healthy human D2R PET images. The pattern of seasonal change was the most consistent in the left caudate, aligning with previous studies showing that dark seasons are associated with lowered amount of dopamine transporter in the left caudate [3]. Also, patients with seasonal affective disorder (SAD) show lowered dopamine transporter binding in the left caudate [30], possibly indicating that dopamine signaling in the left caudate is linked with seasonal onsets of depression symptoms. The effect was also large: one standard deviation of daylength change (i.e., around 4 h) was associated with equal magnitude of D2R binding changes as 2–5 years of ageing in the left caudate. The effect of daylength on D2R was comparable in the datasets with only young and middle-aged adults as well as in the data with full age range. This suggests that the impact of daylength is consistent across age cohorts. Altogether these data suggest that in future studies of D2R binding, especially in high-latitude regions, the effect of seasonality should be considered as it may confound the primary results.

Seasonality in the human brain physiology and particularly neurotransmission remains poorly characterized [31, 32]. There is evidence showing that dark seasons are associated with lowered postsynaptic serotonin receptor availability [33, 34] and probably increased serotonin transporter binding [35, 36]. Clinical in vivo data also indicates a light-sensitive fluctuation in levels of cerebral monoamine oxidase A, an enzyme that degrades neurotransmitters including serotonin and dopamine [37]. Similarly, studies on neuropeptide signaling suggest that the endogenous opioid signaling responds to seasonal rhythms [21, 38]. The present results on dopamine receptor signaling further highlight the brain neurotransmission mechanism on seasonal patterns of cognitive and affective functions.

Previous studies of dopamine signaling using ^18^F-DOPA PET have found an increase in presynaptic synthesis of dopamine during autumn and winter [1, 2]. This complements our study showing increased D2R availability during dark season, signifying that this increase most probably indicates enhanced presynaptic control of dopamine release. D2Rs are known as autoreceptors that modulate the presynaptic synthesis of dopamine [14–16], and the increased D2R signaling is to encounter the increased dopamine synthetic capability. This is also supported by findings that the dopamine transporter binding in the left caudate is lower during dark seasons [3], as D2Rs may not only restrict dopamine release but also enhance transporter functions [17, 18]. Besides, the increased availability of D2Rs may further be mirrored by increased melatonin release during dark seasons, since melatonin is known to inhibit presynaptic dopamine release [39, 40].

However, one study using single photon emission computed tomography with [^123^I]iodo-benzamide to measure dopamine D2/D3 receptor availability, shows that shorter rather than longer sunlight exposure is associated with reduced amounts of striatal receptors [41]. This study used data from low latitude regions where variation of daylength across seasons is small (i.e., around 4 h between longest and shortest days in local region), and the subjects are divided into two groups where unbalanced sex and smoker effects may also complicate the interpretation of the finding. Further, reference tissue modeling of PET data in the study used the frontal lobe as a reference region and this may also compromise their conclusions.

The striatum is a key node for reward responses [42] and energy supply in the caudate may directly affect the feeling of satiation or hunger [43]. Given the role of striatal dopamine signaling in feeding behavior, enhanced D2R signaling in dark seasons may induce overeating [12]. Also, while this effect was observed in both hemispheres, larger effect in the left caudate may hint at a season-dependent lateral difference in the normal brain function, mirroring findings that patients with SADs show state-dependent lateral differences of EEG power and coherence during depressive episodes and following light-induced and summer remission [44]. Only a few studies have focused on the role of dopamine signaling in SADs [30], and therefore, it is challenging to interpret how malfunction in a signal component of this signaling pathway predispose specific symptom such as the overeating character of SAD. Seasonal rhythms profoundly impact mood, with negative affect such as depression, anger, and hostility at lowest rate during the summer [45], whereas symptoms of SAD peak during the winter months [46]. These changes are probably mediated by slow phasic changes in a variety neuroreceptor systems including the dopamine receptor signaling.

### Limitations

The [^11^C]raclopride *BP*_*ND*_ in a baseline condition is proportional to D2R density, but the exact contributions of D2R density, receptor affinity, and baseline occupancy by endogenous dopamine cannot be assessed in a single measurement. The study was based on historical data, where each subject was imaged only once; quasi-experimental design (i.e., natural changes in daylight) was used as longitudinal multi-scan studies would yield a significant radiation load, as in some studies [47, 48]. The data were sampled from different projects and scanners; partial volume effect might contribute to the results causing spillover effects from the adjacent striatal regions, particularly concerning scanners with lower spatial resolutions. Potential scanner-related biases were, however, accounted for in the analyses. Daylength was regarded as a noise-free estimate of local seasons. Day-to-day variance of sunlight exposure and contribution of other seasonal factors (e.g., atmospheric pressure, temperature), however, were not investigated. Longer daylengths in certain seasons generally correspond to more daylight hours, providing people with additional opportunities for outdoor activities and exposure to sunlight. Further, the database was compiled from historical scans and relevant behavioral and self-report measures, such as mood and eating habits, were not systematically collected. Finally, the present findings from high-latitude regions may not generalize to regions with lower latitudes, considering the large magnitude of local photoperiodic variation.

## Conclusions

We conclude that striatal D2R availability is increased during dark seasons. While the exact mechanisms and subsequent impacts of this seasonal pattern of receptor signaling cannot be resolved in this cross-sectional study, our study nevertheless reveals brains’ physiological adaption to seasons at the level of single neurotransmitter system. Considering the important role of striatal dopamine signaling in reward-related behaviors, elevated D2Rs may contribute to the elevated food seeking during dark seasons.

### Electronic supplementary material

Below is the link to the electronic supplementary material.

Supplementary Material 1

## Data Availability

As per Finnish legislation, the medical imaging data as considered to have sensitive personal information cannot be publicly shared even in anonymized format. Enquiries of dataset can be sent to Lauri Nummenmaa by email to latanu@utu.fi or post to Turku PET Centre c/o Turku University Hospital, Kiinamyl-lynkatu 4–8, FI-20520 Turku, Finland.
