# Supplementary material for: Seasonal variation in D2/3 dopamine receptor availability in the human brain
Source: Eur J Nucl Med Mol Imaging. 2024 May 11;51(11):3284–91. doi: 10.1007/s00259-024-06715-9 (PMC11369044; doi:10.1007/s00259-024-06715-9)
Supplement: Supplementary file 1 — Supplementary Material 1 [file 259_2024_6715_MOESM1_ESM.pdf]

Supplementary data

Seasonal variation in D2/3 dopamine receptor availability in the human brain

Lihua Sun[1,2,3], Tuulia Malén[2,3], Jouni Tuisku[2,3], Valtteri Kaasinen[4,5], Jarmo A. Hietala[2,3,6], Juha Rinne[2,3], Pirjo Nuutila[2,3,7], Lauri Nummenmaa[2,3,8]

1. Huashan Institute of Medicine, Huashan Hospital, Fudan University, Shanghai, China
2. Turku PET Centre, University of Turku, Turku, Finland
3. Turku PET Centre, Turku University Hospital, Turku, Finland
4. Clinical Neurosciences, University of Turku, Turku, Finland
5. Neurocenter, Turku University Hospital, Turku, Finland
6. Department of Psychiatry, University of Turku and Turku University Hospital, Turku, Finland
7. Department of Endocrinology, Turku University Hospital, Finland
8. Department of Psychology, University of Turku, Turku, Finland

Corresponding to:
Lihua Sun, lihua.sun@utu.fi
Turku PET Centre, University of Turku
Turku, Finland

Supplementary Table S1. Physical properties have been measured at different times (years 1988-2020) and the measurement standards have slightly changed. The default for radial and tangential average, as well as axial resolution is 1 cm offset. [a] Average resolution (mm FWHM), [b] R0= centre of field of view, [c] Result is in [kcps/kBq/ml] NECR. FWHM= Full width half maximum; FOV= Field of view, FBP= filtered back projection. Reference articles are listed for the data.

Scanners	S1 (Ecat 931)	S2 (GE Advance)	S3 (HRRT)	S4 (HR+)	S5 (VCT PET CT)	S6 (690 PET CT)
Radial and tangential [a]	10.8 [1]	5	2.5	4.4 [2]	5	4.7 [3]
Axial [a]	10.8 [1]	6.6	2.5	5.1 [2]	4.4 [4]	4.74 [3]
Crystal material	BGO	BGO [5]	LSO, GSO [6]	BGO	BGO [4]	LYSO
Crystal size (mm^3)	6.1x12.5x30	4.0x8.1x30 [5]	2.1x2.1x7.5 [6]	4.2x4.39x30	4.7x6.3x30	4.2x6.3x25
Sensitivity (cps/kBq)	6	6.41	39.8 [c]	6.65	8.8 [b] [4]	7.4
Axial FOV	108 [1]	152 [5]	252 [6]	155 [7]	157 [4]	157 [3]
Patient port diameter (mm)	600 [1]	590 [5]	350 [6]	562 [7]	700 [4]	700 [3]
Time-Of-Flight	No	No	No	No	No	Yes
Depth of interaction	No	No	Yes	No	No	No
Reconstruction	FBP	FBP [5]	FORE/HOSP/FBP	FBP, PROMIS [7]	FBP, 2D-3D OSEM	PSF, 3D-OSEM
Attenuation correction method	Ge-68 filled portable ring source	Ge-68 rotating rod source	Cs-137 point source	Ge-68 rotating rod source	Computed tomography	Computed tomography

Supplementary Table S2. Injection information (mean, standard deviation, range) of the radiochemical purity, activity, molar activity, and product mass in the sample. Information is available for 195 out of the total 291 scans, and is presented separately for the studies using single-bolus and bolus-infusion. Note that for some scanners (HRRT and HR+), the injected activity is significantly higher compared with other scanners, which explains the high range and variation in injected activity. MBq= megabecquerel, nmol= nanomol, ug = microgram.

	**Bolus (n= 142)**	**Bolus + infusion (n= 53)**	
Radiochemical purity (%)	99.3, 0.4, 98.1-99.9	99.3, 0.24, 98.7-99.7	
		Bolus	Infusion
Activity (MBq)	238, 76, 119-538	238, 34, 134-285	247, 37, 158-314
Molar Activity (MBq/nmol)	186, 242, 5-1466	338, 228, 8-1483	330, 225, 8-1457
Product Mass (ug)	2.1, 3.0, 0.05-19.6	0.5, 1.5, 0.06-11.3	0.6, 1.6, 0.06-11.6

Supplementary Table S3. Basic information of the primary sample.

**Scanner Types**	**Sex**	**Age**				**Daylength**				**N**
		mean	*s.d.*	*min*	*max*	*mean*	*s.d.*	*min*	*max*	
S1	M	24.62	5.11	19.24	34.68	14.8	3.08	9.12	19.66	13
S2	M	26.8	5.12	19.43	38.75	14.34	3.89	7.73	22.5	61
S3	M	23.58	3.25	19.22	36.17	13.23	3.99	7.71	23.25	88
S4	M	22.9	4.07	18.87	35.58	10.76	3.3	7.79	22.89	18
S5	M	27.46	5.39	20.55	37.03	14.9	5.47	7.7	22.9	15
S1	F	31.06	9.03	19.47	39.47	14.33	6.17	8.3	22.76	8
S2	F	26.28	3.36	20.1	30.33	15.19	6.16	8.53	23.08	8
S3	F	26.79	2.49	25.07	32.69	14.79	5.07	9.93	22.51	8
S4	F	18.82	-	18.82	18.82	11.88	-	11.88	11.88	1
S6	F	26.94	6.34	19.82	39.12	10.83	3.1	8.19	17.49	7

N = number of subjects, M = male, F = female, s.d. = standard deviation, min = minimum, max = maximum

Supplementary Table S4. Basic information of the full sample.

Scanner Types	Sex	Age				Daylength				N
		mean	*s.d.*	*min*	*max*	*mean*	*s.d.*	*min*	*max*	
S1	M	40.14	19.65	19.24	78.4	14.95	3.84	9.12	23.09	23
S2	M	31.89	10.83	19.43	57.78	14.4	4.03	7.72	22.5	78
S3	M	23.58	3.25	19.22	36.17	13.23	3.99	7.71	23.25	88
S4	M	22.9	4.07	18.87	35.58	10.76	3.3	7.79	22.89	18
S5	M	29.57	7.83	20.55	47.12	14.34	5.4	7.7	22.9	17
S1	F	48.76	17.05	19.47	81.62	15.84	5.38	8.3	23.28	21
S2	F	40.64	16.25	20.1	70.76	13.97	5.41	8.53	23.13	16
S3	F	30.26	10.67	25.07	58.02	14.39	4.89	9.93	22.51	9
S4	F	18.82	NA	18.82	18.82	11.88	NA	11.88	11.88	1
S6	F	42.2	13.02	19.82	58.67	13.08	5.13	8.19	22.92	20

N.B., N = number of subjects, M = male, F = female, s.d. = standard deviation, min = minimum, max = maximum

Supplementary Table S5. Effect of daylength on regional D2R availability in the full sample (uncorrected for multiple comparison).

Hem	Region	Beta	95% CI	t	p
Left	Caudate	-0.018	-0.031, -0.0051	-2.74	0.0066**
Right	Caudate	-0.014	-0.027, -0.00012	-1.97	0.049*
Left	Putamen	-0.0077	-0.019, 0.0034	-1.35	0.18
Right	Putamen	-0.010	-0.022, 0.00095	-1.79	0.074
Left	NACC	-0.013	-0.028, 0.0019	-1.70	0.090
Right	NACC	-0.011	-0.027, 0.0044	-1.40	0.16
Left	Thalamus	-0.010	-0.033, 0.012	-0.91	0.37
Right	Thalamus	-0.0036	-0.024, 0.016	-0.36	0.72

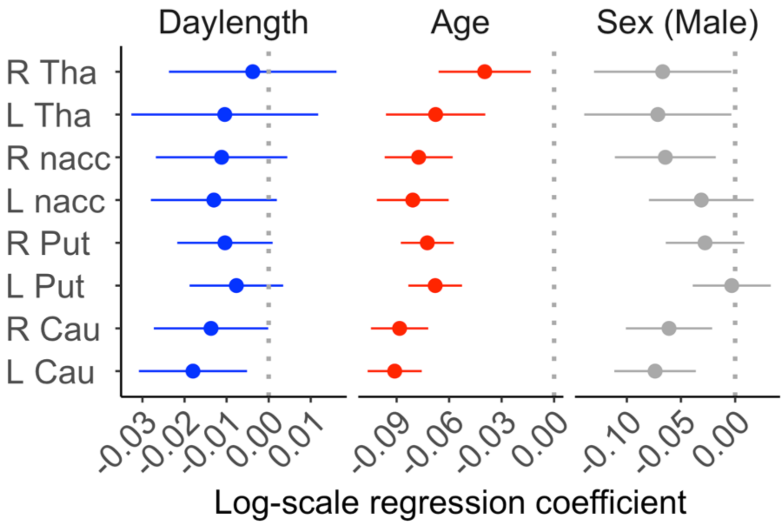

Supplementary Figure S1. Effect sizes of daylength, age and sex (male) on D2R BP_{ND} in different brain regions in the full sample. L = Left, R = Right, Cau = Caudate, Put = Putamen, nacc = Nucleus accumbens, Tha = Thalamus.

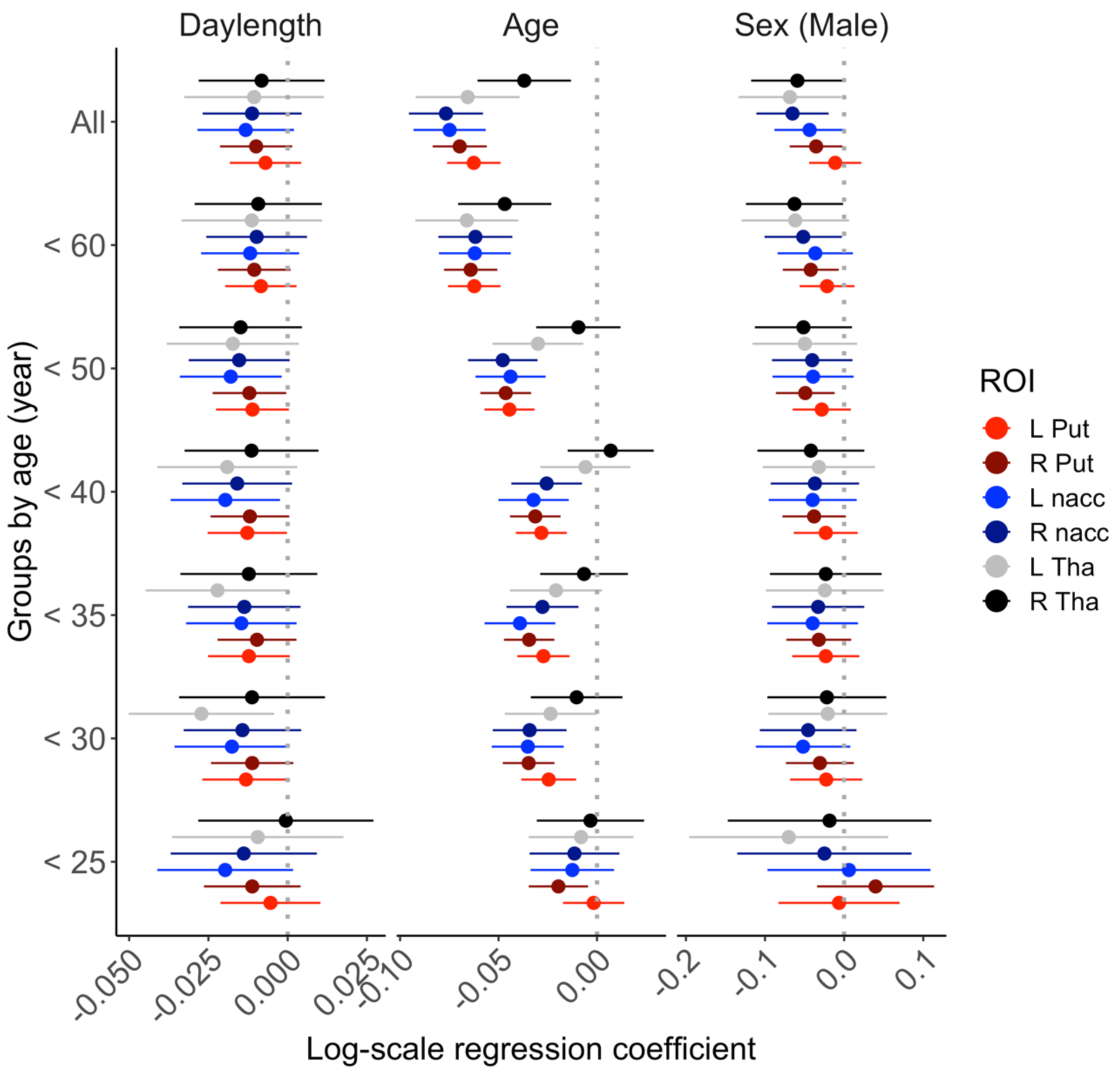

Supplementary Figure S2. Effect sizes for daylength, age and sex (male) on D2R BP_{ND} in different groups defined by maximum age. ROI = region of interest.

References

1. Dahlbom M, Hoffman E, Hoh C, Schiepers C, Rosenqvist G, Hawkins R, et al. Whole-body positron emission tomography: Part I. Methods and performance characteristics. Journal of Nuclear Medicine. 1992;33:1191-9.
2. Herzog H, Tellmann L, Hocke C, Pietrzyk U, Casey ME, Kuwert T. NEMA NU2-2001 guided performance evaluation of four Siemens ECAT PET scanners. IEEE Transactions on Nuclear Science. 2004;51:2662-9.
3. Bettinardi V, Presotto L, Rapisarda E, Picchio M, Gianolli L, Gilardi M. Physical Performance of the new hybrid PET/CT Discovery-690. Medical physics. 2011;38:5394-411.
4. Teräs M, Tolvanen T, Johansson J, Williams J, Knuuti J. Performance of the new generation of whole-body PET/CT scanners: Discovery STE and Discovery VCT. European journal of nuclear medicine and molecular imaging. 2007;34:1683-92.
5. DeGrado TR, Turkington TG, Williams JJ, Stearns CW, Hoffman JM, Coleman RE. Performance characteristics of a whole-body PET scanner. J Nucl Med. 1994;35:1398-406.

6. Schmand M, Eriksson L, Casey M, Andreaco M, Melcher C, Wienhard K, et al. Performance results of a new DOI detector block for a high resolution PET-LSO research tomograph HRRT. IEEE Transactions on Nuclear Science. 1998;45:3000-6.
7. Brix G, Zaers J, Adam LE, Bellemann ME, Ostertag H, Trojan H, et al. Performance evaluation of a whole-body PET scanner using the NEMA protocol. National Electrical Manufacturers Association. J Nucl Med. 1997;38:1614-23.
